# Incorporating DEM Uncertainty in Coastal Inundation Mapping

**DOI:** 10.1371/journal.pone.0108727

**Published:** 2014-09-24

**Authors:** Javier X. Leon, Gerard B. M. Heuvelink, Stuart R. Phinn

**Affiliations:** 1 Global Change Institute, The University of Queensland, Brisbane, Queensland, Australia; 2 School of Geography, Planning and Environmental Management, The University of Queensland, St Lucia, Australia; 3 Soil Geography and Landscape group, Wageningen University, Wageningen, The Netherlands; University California Los Angeles, United States of America

## Abstract

Coastal managers require reliable spatial data on the extent and timing of potential coastal inundation, particularly in a changing climate. Most sea level rise (SLR) vulnerability assessments are undertaken using the easily implemented bathtub approach, where areas adjacent to the sea and below a given elevation are mapped using a deterministic line dividing potentially inundated from dry areas. This method only requires elevation data usually in the form of a digital elevation model (DEM). However, inherent errors in the DEM and spatial analysis of the bathtub model propagate into the inundation mapping. The aim of this study was to assess the impacts of spatially variable and spatially correlated elevation errors in high-spatial resolution DEMs for mapping coastal inundation. Elevation errors were best modelled using regression-kriging. This geostatistical model takes the spatial correlation in elevation errors into account, which has a significant impact on analyses that include spatial interactions, such as inundation modelling. The spatial variability of elevation errors was partially explained by land cover and terrain variables. Elevation errors were simulated using sequential Gaussian simulation, a Monte Carlo probabilistic approach. 1,000 error simulations were added to the original DEM and reclassified using a hydrologically correct bathtub method. The probability of inundation to a scenario combining a 1 in 100 year storm event over a 1 m SLR was calculated by counting the proportion of times from the 1,000 simulations that a location was inundated. This probabilistic approach can be used in a risk-aversive decision making process by planning for scenarios with different probabilities of occurrence. For example, results showed that when considering a 1% probability exceedance, the inundated area was approximately 11% larger than mapped using the deterministic bathtub approach. The probabilistic approach provides visually intuitive maps that convey uncertainties inherent to spatial data and analysis.

## Introduction

The 0.2 m of approximate global sea level rise (SLR) over the past 100 years is the result of higher global temperatures due to increased atmospheric CO_2_ levels. This rise is forecast to accelerate during the next decades, reaching a level of about 1 m or more by 2100, potentially resulting in the permanent inundation of large areas of low-lying coastal land [1], [2]. Globally, it has been estimated that between 9.2 and 10.9% of the world population in 2006 lived on land areas adjacent to the sea and 10 m below mean sea level [3]. In addition to coastal inundation, extreme high sea levels and associated erosion driven by meteorological events such as storms and cyclones, changes in wave climate and potential changes in regional El Niño–Southern Oscillation (ENSO) are also forecasted to increase in magnitude [4]–[6].

The physical, ecological and socio-economic impacts of SLR are of great concern [7]–[9] but the complexity and uncertainties inherent to this issue increases the challenges of implementing effective adaptation policies [1]. Coastal planners, decision-makers and stakeholders require reliable spatial data on the extent and timing of potential coastal inundation and associated hazards to better manage related risks. Most SLR vulnerability assessments are elevation-based, where areas adjacent to the sea and below a given elevation (e.g. representing an SLR forecast or storm surge level) are mapped using a deterministic line dividing potentially inundated from dry areas [10]. This approach, known as the bathtub method, is easily implemented and hence commonly used, only requiring an elevation dataset usually in the form of a digital elevation model (DEM) [11].

The quality of the DEM, which is a function of the spatial resolution and vertical accuracy of the data source, has a great influence on the accuracy of the inundation mapping [12]. Airborne light detection and ranging (LiDAR) has become the most cost-effective and efficient technique to collect terrain data over large areas and generate high-resolution, high-accuracy DEMs [13]. LiDAR-derived DEMs have typical horizontal spatial resolutions of 1 m and a global vertical accuracy better than 0.2 m, making them suitable to depict subtle features on otherwise flat coastal areas. Evidence shows that resolving fine objects such as roads and other infrastructure results in a better delineation of coastal inundation and more reliable vulnerability assessments [12], [14]. However, quantifying the accuracy of inundation mapping cannot be based only on summary measures of vertical accuracy. The error in a DEM, defined as the difference between predicted and measured ground elevation (hereinafter referred to as elevation errors), is spatially variable and cannot be adequately represented with a single global parameter of dispersion, such as the commonly used root mean square error (RMSE) [15]. For example, measured ground or bare-earth LiDAR elevations can be considerably less accurate depending on land cover type such as dense vegetation [16], [17], or terrain variables such as slope, convexity or ruggedness [18]–[21]. In addition, error is added during the data interpolation process [22], [23], propagating uncertainties into the final mapping. Elevation errors are also spatially correlated, meaning that error values tend to be similar at nearby locations, and this can also have a considerable impact on the output of spatial analyses that use a DEM as input.

Uncertainty is inherent to spatial data and spatial analysis and therefore it is of paramount importance to effectively communicate it, particularly when dealing with decision-making in a changing climate. Communicating uncertainty in terms of probability distributions to a diverse audience is challenging but becoming more popular and effective with the use of visualization techniques (e.g. for weather, sports, economics) [24]. In the context of sea-level rise mapping, few studies have attempted to communicate, in a spatially-explicit way using maps, the effects of elevation errors and uncertainty in the final vulnerability assessments (i.e. uncertain location of the inundation line). Notable exceptions include the work by Gesch [11] and Gesch [25] where uncertainty was incorporated in maps of potential SLR inundation. Elevation error was characterised in these studies by calculating the RMSE of the elevation dataset. The RMSE was treated as the standard deviation from which confidence intervals can be derived at different confidence levels assuming unbiased predictions and a Normal distribution. The linear error with a 95% confidence level (LE95), a metric used by the National Standard for Spatial Data Accuracy (NSSDA) [26], was calculated by:
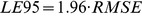
(1)


However, assumptions required for using the RMSE to characterise the elevation error, such as the stationarity of the variance, are disputed [15], [22]. Acknowledging these limitations, Gesch [25] highlighted the importance of future research examining the effects of the spatial variability of elevation errors in sea-level rise vulnerability assessments.

More recently, Schmid et al. [27] extended the deterministic linear error approach to a probabilistic approach by calculating and mapping z-score values based on the RMSE of the elevation data. In addition, the approach incorporated uncertainties associated with determining water surfaces such as shifting tidal datum. Furthermore, Cooper and Chen [28] evaluated the effect of combining vertical uncertainties in elevation data, datum transformation and future SLR estimates using Monte Carlo simulation to propagate probability distributions through the inundation models. Even though both approaches considerably improved mapping and portraying inundation uncertainty in bathtub-derived SLR vulnerability assessments, the underlying assumption of unbiased, uncorrelated and stationary errors in the elevation data remains a shortcoming.

The overall aim of this study was to assess the impacts of spatially variable and spatially correlated elevation errors in high-spatial resolution DEMs when applied to the mapping of coastal inundation. In order to account for the spatial variability of elevation errors and the propagation of uncertainty to bathtub-derived inundation maps, we used a probabilistic approach based on geostatistical simulations that generates the input required for a Monte Carlo uncertainty propagation analysis. This approach effectively communicates uncertainty using visually intuitive maps that can support decision-making. Accounting for uncertainties in other components of SLR vulnerability assessments, such as sea-level forecasts [28], determination of future water level [27] or estimation of population at-risk [29], although very important, but are not part of the scope of the present study.

## Methods

### Study area

**Figure 1:**
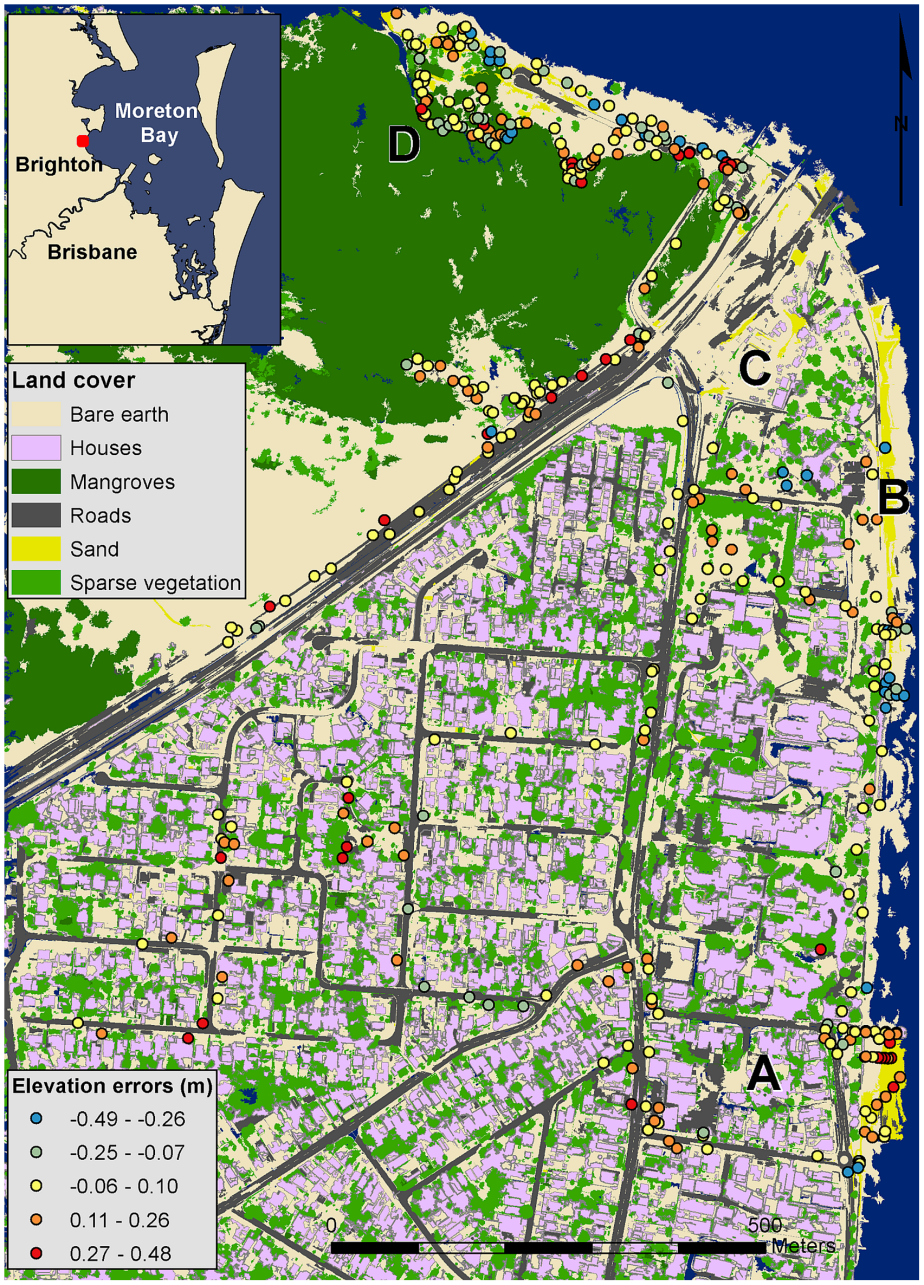
Location of study area. Classified land cover map with overlaid elevation errors for the 407 field measurements collected with real time kinematic GPS. Representative land covers for the study area: (A) low-lying suburban housing, (B) low energy sandy beaches and (C) low-lying open spaces (D) to the south of the highway and wetlands (D) to the north.

**Figure 2:**
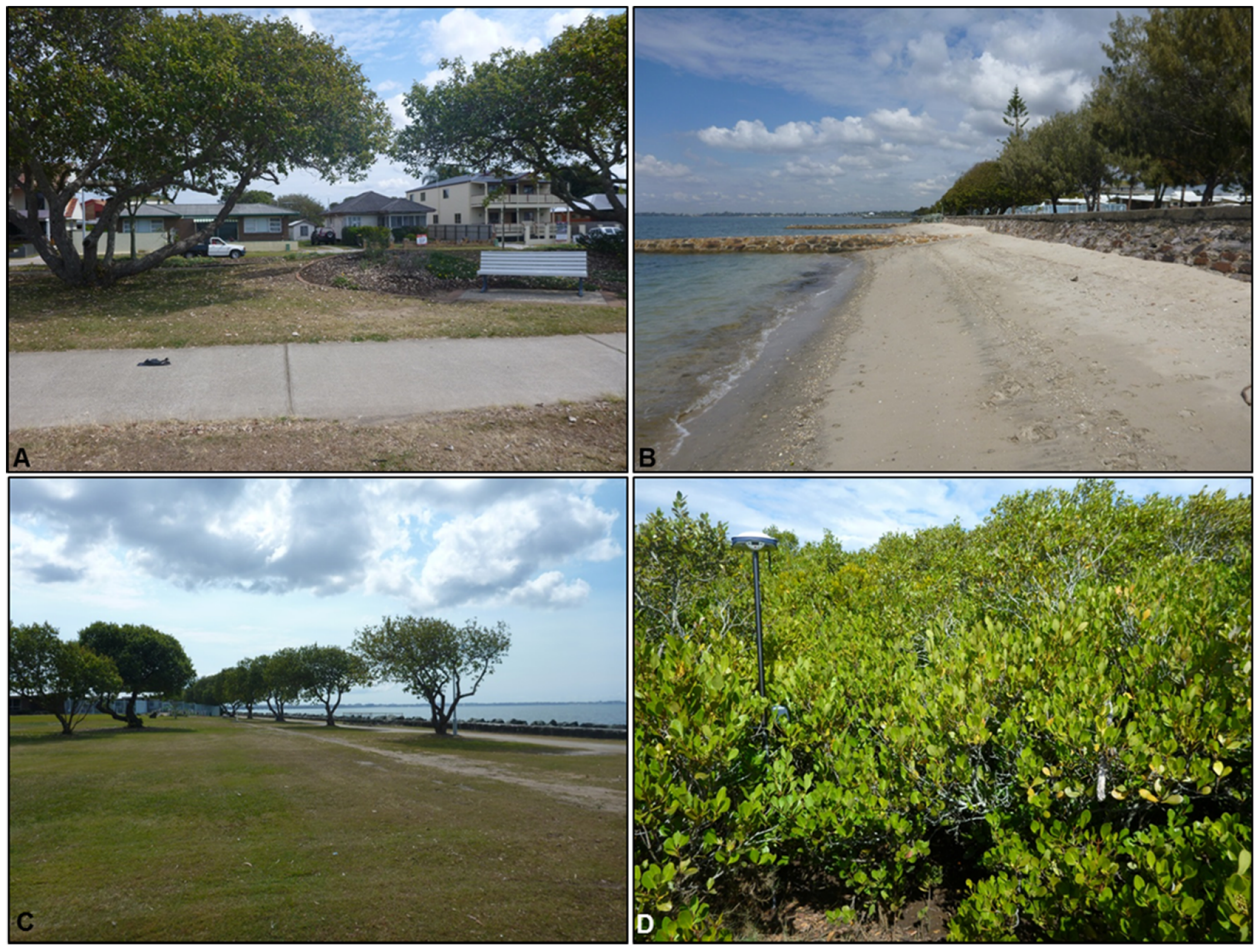
Representative land covers for the study area. (A) Low-lying houses, (B) low-energy sandy beaches, (C) low-lying bare earth/open spaces and (D) mangroves forest (Source: Javier X. Leon).

The study area covers approximately 1.5 km^2^ of Brighton, the northernmost suburb of Brisbane City, Australia. Brighton is located 19 km north of the Brisbane city centre and features mostly suburban housing on low-lying coastal land adjacent to Moreton Bay (Figure 1). The area has different land covers and infrastructure that are prone to inundation due to storm surge and future rises in sea level [30]. These include low-lying houses, low-energy sandy beaches, parks and roads infrastructure and wetlands including mangroves fringing the coast (Figure 2). The prevailing low-energy conditions in the mesotidal (approximately 2 m), semi-enclosed, estuarine Moreton Bay are amenable to bathtub mapping, as erosion is not the most significant process.

### Field data

The suburb of Brighton (longitude 153.061613, latitude −27.288925) was visited on two occasions (22 August 2012 and 13 October 2013). During both visits real time kinematic (RTK) GPS measurements were surveyed with nominal precisions of <2 cm in the horizontal and <5 cm in the vertical positions. No specific permissions were required for the topographic surveys on the visited locations and the field studies did not involve endangered or protected species. The horizontal coordinate system utilized was the Australia (MGA) Zone 56, Geocentric Datum Australia 1994 (MGA56-GDA94). Two benchmarks installed by the Brisbane City Council using spirit levelling (3rd Order, Class C vertical control) were used to install the RTK GPS base antenna and reduce the ellipsoid heights to Australian Height Datum (AHD), which for this area closely corresponds to mean sea level (MSL) [30]. The survey was closed by undertaking a reverse base-station and rover traverse (<0.03 m).

A total of 407 points (108 and 299 in 2012 and 2013, respectively) were measured, mostly along easily accessible transects (i.e. roads, beaches, paths) across the major land cover areas and areas with different topographical characteristics (slope, ruggedness) (Figure 1). Orthophoto mosaics from 2009 and 2012 (see Section 2.3) were used to identify and avoid areas where land cover had considerably changed during the period of LiDAR data acquisition in 2009 (see Section 2.4) and the RTK GPS field data surveying.

### Remotely-sensed imagery

Two remotely-sensed image datasets were used in this study. The first dataset was a very high resolution (7 cm spatial resolution) orthophoto mosaic derived from true-colour (red, green and blue) aerial photographs (http://maps.nearmap.com). Orthophoto mosaics covering the study area in 2009 and 2012 were used to evaluate areas where considerable changes occurred during the period of LiDAR data acquisition in 2009 (Section 2.4) and RTK GPS field data surveying in 2012 (Section 2.2). The 2012 orthophoto mosaic was georeferenced using five ground control points measured with RTK GPS (RMSE 0.08 m). The 2009 orthophoto mosaic was georeferenced to the 2012 mosaic (RMSE 0.047 m). Both orthophoto mosaics were resampled to 1 m resolution.

The second dataset was a Quickbird high-spatial resolution satellite image (2.4 m spatial resolution) obtained in August 2010. The image was sub-sampled to 1 m spatial resolution to match the resampled orthophoto mosaics. A natural neighbours resampling algorithm was used in order to honour the original range of values, while keeping a smooth surface avoiding peaks or pits not represented by the samples [31]. The Quickbird image was georeferenced to the 2012 orthophoto mosaic (RMSE 1.1 m). Lastly, the well-known normalized difference vegetation index (NDVI) was calculated from the image:
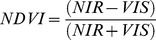
(2)where NIR and VIS stand for the spectral reflectance measurements acquired by the Quickbird sensor in the near-infrared (Band 4) and visible (red) (Band 3) regions, respectively.

A land cover map was derived from the 2012 orthophoto mosaic, the Quickbird image and the LiDAR dataset (Section 2.4) following an object-based image analysis (OBIA) approach similar to Arroyo et al. [32]. The advantage of employing this approach was that the performance of the classification improved by incorporating spectral information (e.g. NDVI), geometric characteristics (e.g. rectangular fit of houses), contextual rules (e.g. beach adjacent to water) and terrain variables (e.g. slope). The classes mapped were: bare earth, houses, mangrove forest, roads, sandy beach and sparse vegetation.

### Terrain data

A LiDAR dataset acquired by the State of Queensland’s former Department of Environment and Resource Management (DERM) was used in this study. The LiDAR data were collected for the South-East Queensland Priority Area from a fixed wing aircraft during ten flights between March 25th and April 24th, 2009. Acquisition was undertaken within +/− 2 hours of low tide to ensure maximum coverage over tidal flats. LiDAR points (approximately 1.5 points/m^2^) were filtered and classified as ground and non-ground results. Ground points were interpolated to a high-resolution 1 m topographic DEM using the natural neighbour algorithm. Data acquisition and post-processing was controlled to achieve a vertical accuracy (RMSE) within 0.15 m and horizontal accuracy within 0.45 m (RMSE). The MGA56-GDA94 coordinate system was used for the horizontal datum projection and AHD for the vertical datum [33].

The DEM was further smoothed using a low-pass filter (3×3 m window) to remove remaining outliers and prepare the data for subsequent terrain analysis [34]. Terrain variables were derived using SAGA 2.0 GIS software [35]. These included: slope, aspect, profile and plan curvature, mean curvature [36], terrain convexity [37], topographic position index (TPI) [38], terrain ruggedness index (TRI) [39], multiresolution index of valley bottom flatness (MRVBF) [40] and vector ruggedness measure (VRM) [41].

### Error propagation

**Figure 3:**
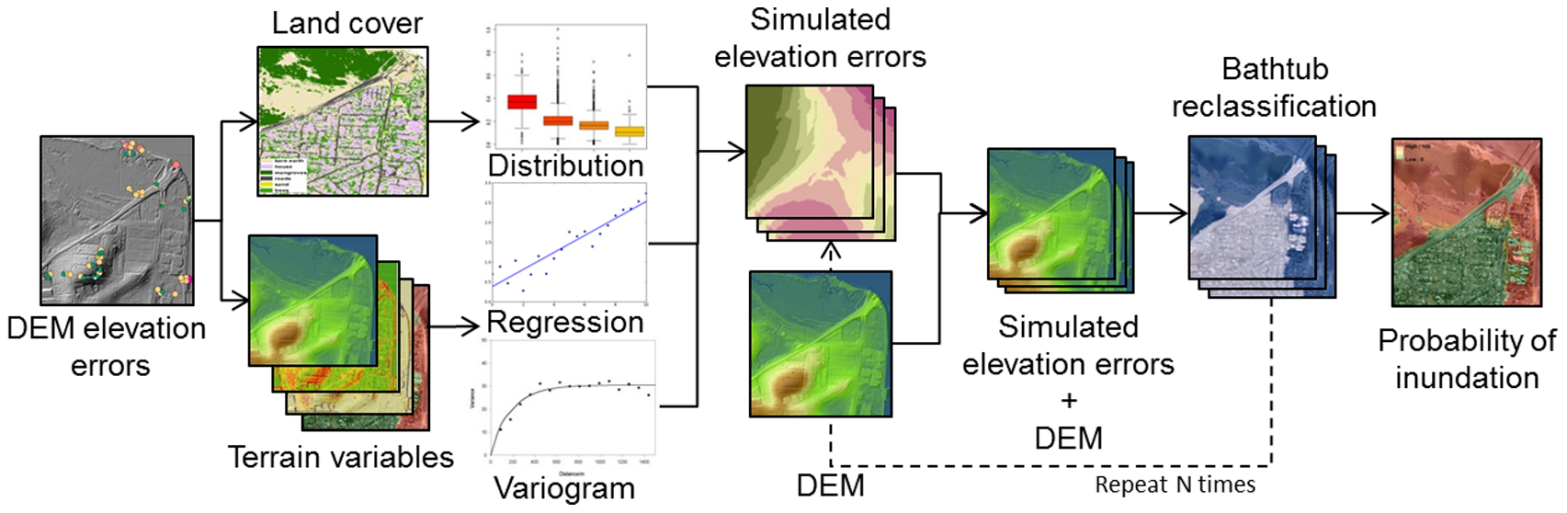
Flowchart of uncertainty propagation analysis.

A spatial stochastic model-based method was used to incorporate the propagation of errors in the LiDAR-derived DEM to bathtub SLR inundation maps. This approach offers a basis for incorporating and handling uncertainty in spatial modelling, but to date it has not been widely adopted [42], with some exceptions [43]–[45]. As Hengl et al. [43] noted, spatial error propagation analysis is beneficial when errors are spatially variable and spatially correlated. A general workflow of the utilized approach is presented in Figure 3.

Elevation errors in the LiDAR-derived DEM were assessed using the more accurate RTK GPS surveyed points by subtracting the RTK GPS measured points from the DEM. The distribution of elevation errors was explored for normality and correlation with land cover and terrain variables. In order to simulate the random component of the elevation error a Monte Carlo simulation based on geostatistics was used. Sequential Gaussian simulation (SGS) was chosen as it is the most common technique to generate conditional stochastic simulations for spatially continuous variables [46]. SGS uses the geostatistical interpolator kriging to simulate values. Kriging is a generic name for a family of generalised least-squares regression algorithms based on regionalized variable theory that assumes that spatial variation of the variable is statistically homogeneous throughout the region. Kriging, as opposed to deterministic interpolators, utilizes a model of the spatial correlation or structure of processes. The spatial structure is characterised by a variogram, which is estimated from the sampled data. The variogram is then used to estimate kriging weights used for data interpolation. Under the assumptions made, kriging is an optimal spatial interpolation method (i.e. predictions are unbiased with minimum prediction error variance). It also allows to quantify prediction accuracy with the kriging variance [47].

Briefly, SGS works by randomly selecting a location (i.e. grid cell) from the spatial domain. At this location a conditional probability distribution of the elevation error is computed using, for example, ordinary kriging, a simple and effective kriging method that assumes a constant mean. Next a value is randomly drawn from this probability distribution using a pseudo-random number generator and assigned to the location. The simulated value is added to the dataset and a new location is randomly selected. The process is repeated until all locations have been visited, each time including all previously simulated values in order to preserve the spatial correlation in simulated values, as modelled by the variogram.

More accurate simulations of topography are produced by including both the deterministic and the spatially correlated random components of the elevation errors. For example, regression-kriging instead of ordinary kriging [45]. Regression-kriging treats the variable of interest (i.e., the elevation error) as the sum of a deterministic trend and a zero-mean stochastic residual. The trend is often taken as a linear combination of exhaustively known environmental variables, such as land cover and terrain. The estimated trend is added to the kriged stochastic residual [48]. The advantage of regression-kriging is that it allows a separate interpretation of the deterministic and random components of spatial errors and extends the method to a broader range of regression techniques [49]. Despite some limitations [49], [50], regression-kriging generally outperforms other spatial interpolation methods [51]–[54]. Regression-kriging predictions are calculated by:
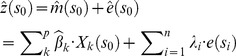
(3)where 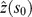 is the predicted value, 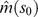 is the fitted linear regression and 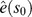 is the spatially interpolated residual at the prediction location 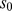. The 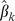 are the estimated regression coefficients and 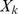 the explanatory variables. 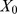 is often taken as unity so that 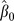 is the intercept of the regression model. The 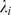 are kriging weights for the residuals, where 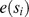 is the residual at measurement location 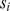 i.e., the difference between the elevation error and the fitted trend at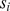. The regression coefficients 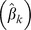 are estimated using generalized least squares (GLS), and hence incorporate the spatial correlation of the stochastic residual of the regression-kriging model. SGS based on the regression-kriging model can also be obtained for spatial stochastic simulation. This is done in the same way as explained before, where the kriging step uses regression-kriging instead of ordinary kriging.

Geostatistical simulations were run using both ordinary kriging and regression-kriging interpolators for comparison purposes. The Exploratory Regression tool, as implemented in ArcGIS 10.2 [55], was used to evaluate all possible combinations of explanatory variables used to model the deterministic trend for the regression-kriging analysis. Exploratory regression is similar to stepwise regression but in addition to looking at high Adjusted R^2^ values, other factors are included to ensure that the requirements and assumptions of a properly specified linear regression are met. Factors include statistically significant coefficients for all explanatory variables, avoidance of multi-colinearity (Variance Inflation Factor <7.5) and normally distributed residuals (non-significant Jarque-Bera probability value) [55]. Environmental variables explaining elevation errors included orthophoto mosaic and Quickbird image spectral bands (Section 2.3), terrain variables (Section 2.4) and land cover class, which was incorporated in the regression analysis as a factor.

The spatial structure of the elevation errors, for both ordinary kriging and regression-kriging models, were modelled using the valid and flexible Matérn variogram [56]. The simulation process was fully performed with the GSTAT package [57] as implemented in the R Environment for Statistical Computing software [58]. A total of 1,000 realizations of elevation errors were generated to guarantee a stable result [59]. Simulated elevation errors were added to the original LiDAR-derived DEM and inundation maps were produced using the bathtub approach following the hydrological connectivity method proposed by Poulter and Halpin [10].

For the sake of simplicity, a scenario of 3.9 m above MSL, a linear combination of a 2.9 m storm surge over a 1 m SLR above MSL, was chosen for the bathtub inundation map, even though in reality the interactions between SLR and storm surge are highly complex [60]. The 2.9 m storm surge water level is equivalent to the 100 year storm average recurrence interval (ARI), or a storm with 1% annual exceedance probability, calculated for this area [30]. Maps of inundation with 1% and 50% probability of exceedance were derived for comparison purposes with the deterministic inundation map.

## Results

### Elevation errors

**Figure 4:**
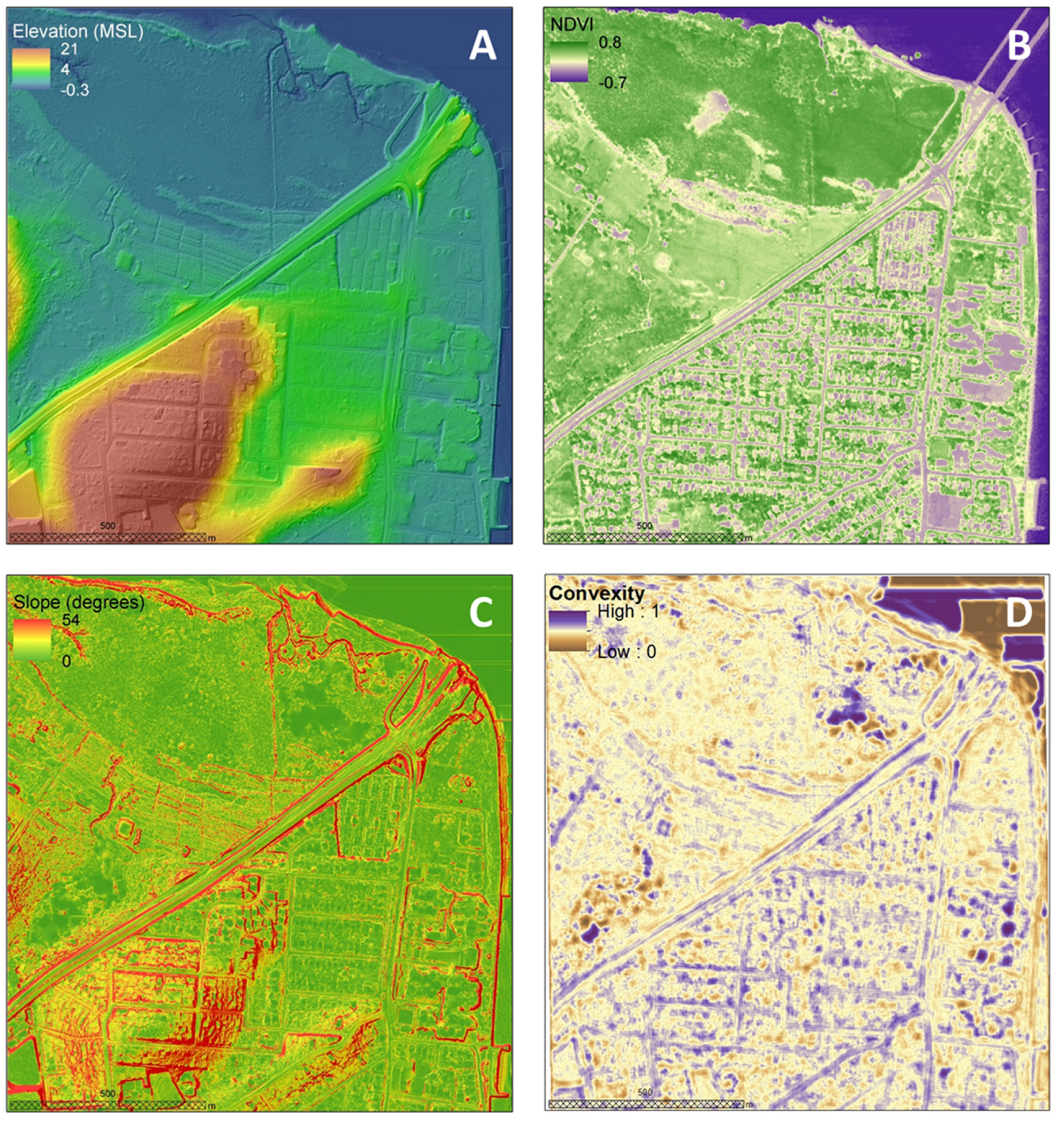
Spatial datasets used in regression analysis. (A) Digital elevation model (DEM), (B) Normalized difference vegetation index (NDVI) and terrain variables including (C) slope and (D) terrain convexity. DEM was based on data provided by the State of Queensland’s former Department of Environment and Resource Management and NDVI was based on satellite image provided by DigitalGlobe.

**Figure 5:**
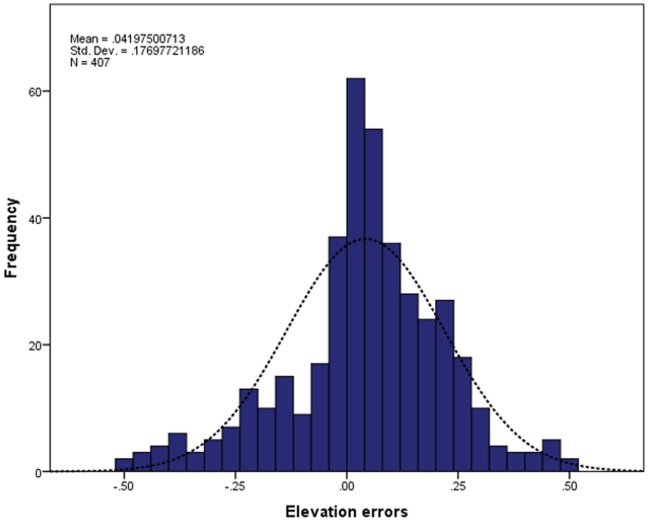
Histogram of elevation errors.

**Figure 6:**
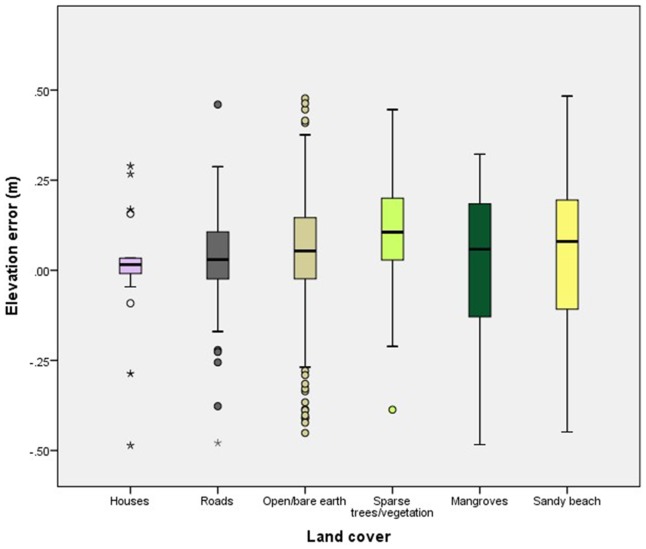
Box-whisker plots of elevation errors grouped by land cover.

The DEM elevations ranged from 0.03 to 21 m above MSL, with an average elevation of 4 m above MSL (Figure 4). Elevation errors, as quantified from the 407 RTK GPS points, closely approximated a normal distribution and varied from −0.49 to 0.48 m. Elevation errors were slightly biased with a mean value of 0.04 m (Figure 5). The standard deviation of 0.18 m was consistent with commonly reported errors in LiDAR-derived DEM. The spatial distribution of elevation errors showed some clustering mainly correlated with land cover (Figure 1). Correlation analysis between elevation errors and land cover revealed that smaller errors were found on “built” and homogeneous environments such as houses, roads and bare earth. The largest elevation errors were found on vegetated areas, including both sparse trees and mangrove, and on sandy beaches. A box plot of elevation errors grouped by land cover is presented in Figure 6.

The deterministic component of the elevation errors was first modelled using OLS regression. The best model incorporated the following significant predictors: land cover, NDVI, slope and convexity (Table 1). The model avoided multicollinearity (VIF = 1.04) and residuals were sufficiently normally distributed (non-significant Jarque-Bera probability values). The predictors explained 14% of the variance in the elevation errors.

**Table 1:** Regression coefficients for best linear model.

	Estimate	Standard Error	t value	Pr(>|t|)	
(Intercept)	0.350	0.077	4.562	0.000	***
NDVI	−0.156	0.051	−3.063	0.002	**
slope	0.514	0.103	4.992	0.000	***
convexity	−0.579	0.113	−5.122	0.000	***
landcover (bare earth)	−0.104	0.045	−2.319	0.021	*
landcover (sparse vegetation)	−0.111	0.053	−2.077	0.038	*
landcover (sand)	−0.156	0.054	−2.877	0.004	**
landcover (mangrove)	−0.065	0.051	−1.276	0.203	
landcover (roads)	−0.054	0.047	−1.166	0.245	

Signif. codes: 0 ‘***’ 0.001 ‘**’ 0.01 ‘*’ 0.05 ‘.’ 0.1 ‘ ’ 1.

The spatial structure of elevation errors showed a range of 102 m and partial sill of 0.023.

**Figure 7:**
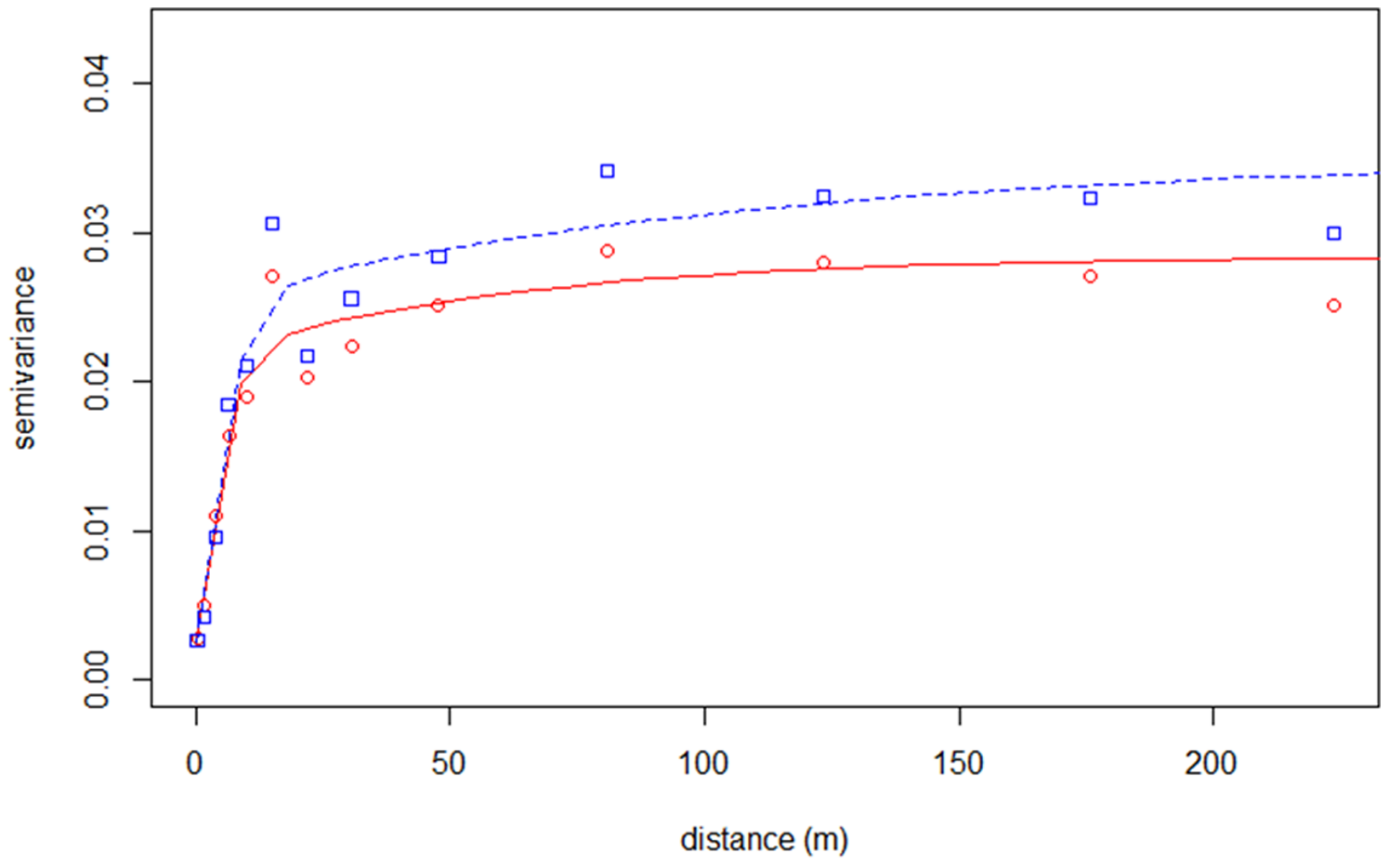
Variograms of elevation errors. Fitted Matérn variogram based on ordinary kriging model (blue dashed line and squares) and regression-kriging model (red line and circles).

Both the range and partial sill of the variogram fitted to GLS-derived residuals were smaller (65 and 0.018, respectively), as expected due to about 14% of variance being explained by the regression model (Figure 7).

### Inundation mapping

**Figure 8:**
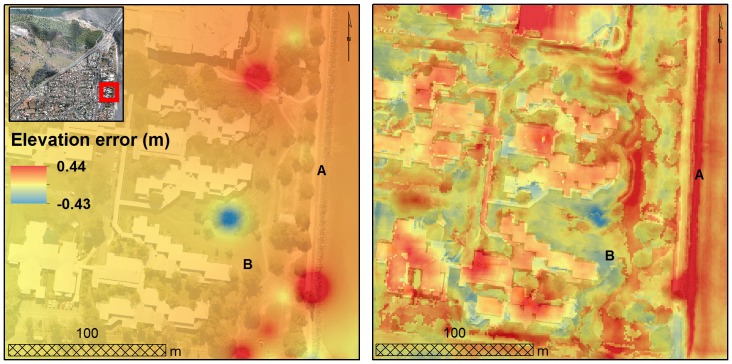
Interpolated elevation errors. Inset showing interpolation elevation errors based on ordinary kriging model (left panel) and regression-kriging model (right panel). Location of (A) seawall and (B) park are shown for reference (see main text).

Interpolated elevation errors based on ordinary kriging and regression-kriging models showed considerable differences across the study area (Figure 8). Interpolation based on the regression-kriging model was visually more realistic than the ordinary kriging-derived interpolation. For example, complex terrain or vegetated areas such as parks were better approximated by the regression-kriging model. The ordinary kriging-derived DEMs underestimated structures such as steep seawalls by up to 20 cm (Location A in Figure 8). Conversely, the elevation of parks was overestimated by up to 15 cm (Location B in Figure 8). Based on this, the regression kriging-derived DEMs were deemed more appropriate for inundation mapping in our study site.

The 1,000 regression-kriging -derived simulated elevation error maps were added to the LiDAR-derived DEM and reclassified using bathtub inundation modelling. The adopted bathtub modelling ensured inundated areas were hydrologically connected to the ocean, thus avoiding flooding of inland depressions not connected to the ocean and potential overestimation of inundated areas. The probability of inundation to a scenario combining a 2.9 m storm surge (ARI100 event) over a 1 m SLR was calculated by counting the proportion of times from the 1,000 simulations that a location was inundated.

**Figure 9:**
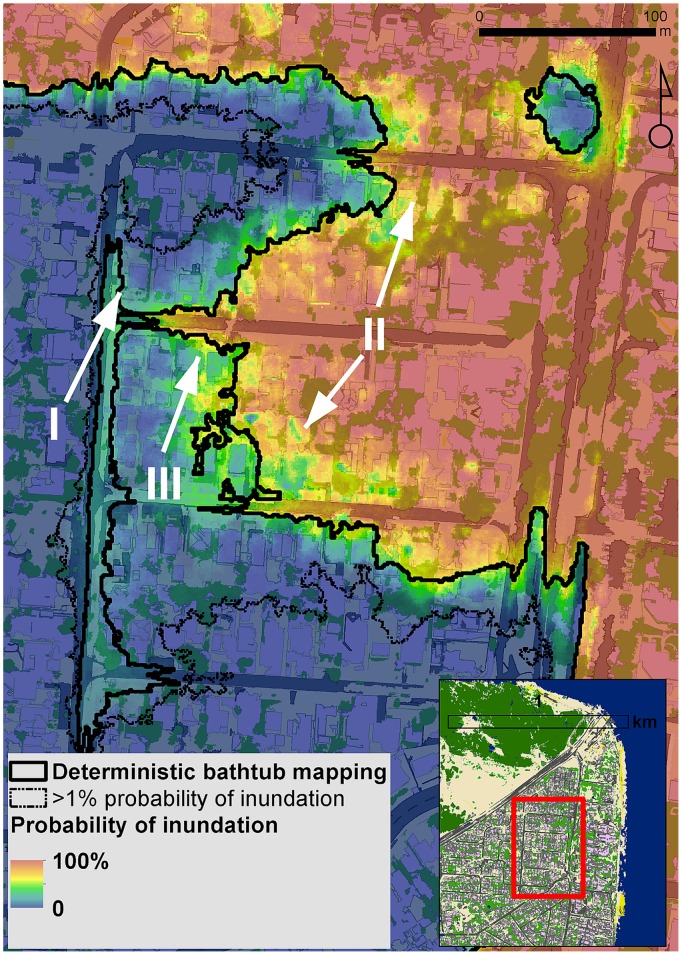
Probability inundation map for a scenario combining a 2.9 m 100 ARI storm surge event over a 1 m SLR. The fat solid black line represents the deterministic bathtub-derived inundation border. The thin solid line shows the area that has a probability greater than 1% to become inundated.

Inundation maps based on the deterministic and geostatistical bathtub approaches are shown in Figure 9. The bathtub method includes non-linear operations and spatial flows which results in a slightly larger area (0.5%) for the inundation map obtained for the determinstic run than the median of the probabilistic inundation map. However, when considering a 1% probability exceedance, the inundated area is approximately 11% larger than that mapped using the deterministic approach. This additional inundated area conveys information about elevation errors and propagation of uncertainties through the mapping and spatial analysis process. For example, low-lying and flat areas such as roads (zone I, Figure 9) or houses surrounded by vegetation (zone II, Figure 9) are mapped as inundated based on the deterministic bathtub map but appear as uncertain areas with lower probability (<30%) of becoming inundated based on the probabilistic mapping. Conversely, areas with houses on complex terrain (zone III, Figure 9) that appear safe from inundation based on the deterministic mapping can have a large probability (60–90%) of getting inundated when considering the propagation of uncertainty through the spatial analysis. Being able to quantify the probability of inundation at any location is of great interest from a risk management perspective.

## Discussion

The geostatistical approach presented in this study accounted for the spatially variable and spatially correlated elevation errors inherent to high-resolution DEMs. The use of SGS dealt effectively with the complexities of analysing uncertainty propagation through the spatial analysis process (e.g. bathtub modelling). Hence, this approach is suitable for the mapping of complex and dynamic phenomena such as inundation due to future SLR, storm surge or a combination of these.

The main limitation of applying this method is related to data constraints. Acquiring the required datasets simultaneously or within a short temporal span is very challenging and therefore uncommon. In this study, LiDAR data were acquired in 2009 while ground control data were acquired three years later. Further, ground control data were surveyed in two separate occasions almost one year apart. Based on visual interpretation of the very high resolution orthophoto mosaics from 2009 and 2012, we can safely assume that most sites surveyed within the study area did not undergo considerable change. Areas that were observed to change considerably, such as the motorway around the bridge, were avoided during the RTK GPS survey. However, even though planform changes on the low-energy sandy beaches were not observed, it is probable that changes in profile shape occurred, resulting in the larger elevation errors obtained for that land cover category. This is important to note, particularly on dynamics coastlines, as SLR vulnerability assessments are rarely based on up-to-date LiDAR elevation datasets.

The quality of datasets is also important for the correct application of the proposed method. The fitting of the regression model and the variogram are essential to the quality of the geostatistical model. These rely on both the quality and distribution of ground control data in the spatial and feature space domains of the explanatory datasets. Highly accurate datasets (e.g. RTK GPS survey) covering large areas are seldom available but are required to assess the spatial variability of DEM errors. This is a challenge for the transferability of this and other empirical statistical approaches. However, based on the findings of this and other studies [16], [23], terrain variables and/or land cover maps could be incorporated in a quantitative analysis of the error in elevation data when ground control data are not available.

Geostatistical simulations have not been widely adopted in geoscience or spatial data analysis applications due to other factors such as computational challenges [61]. The exponential increase in computational power and recent software development [54] will probably change this trend. Furthermore, geostatistical simulations produce DEMs with additional noise by incorporating the complete variability of elevation error, as opposed to the smoother DEMs sought by geomorphologists or specialists performing terrain analysis. However, incorporating additional explanatory datasets within the regression-kriging model produces more realistic DEMs.

It is important to note that geostatistical methods take the spatial correlation in elevation errors into account. This has a great impact on analyses that include spatial interactions such as inundation mapping. For instance, if errors were assumed spatially uncorrelated, then simulations would be very noisy and there would be many ‘barriers’ against flow, resulting in underestimated inundation areas.

The novel application of this technique to the mapping of coastal inundation has two further advantages over the more traditional deterministic mapping. First, it is visually intuitive. Experts and non-experts can equally interpret the probability of some event occurring (e.g. house being inundated), especially as people are increasingly used to dealing with probabilities (chance of rain, sporting bets, elections, etc.). By visualizing the different probabilities of risk to inundation, stakeholders and decision-makers can further visualize adaptation options that can improve communication of policy and public engagement [62].

Second, it explicitly provides information about the reliability of the final inundation maps. This can avoid potential misinterpretation when assessing vulnerabilities due to SLR or storm surge. The line on a deterministic bathtub-derived inundation map delimits an area with approximately 50% chance of being inundated. It is commonly assumed that areas above the line will be safe and those below will not be, despite the various limitations in the vulnerability assessment, hence potentially creating a false sense of security or fear amongst the stakeholders and general public. The substitution of the more traditional deterministic “line on a map” by a probabilistic area explicitly incorporates uncertainties of the final map and can be easily incorporated into risk management and decision-making processes. For example, a risk-aversive decision making approach can be taken by planning for scenarios where the probability of inundation exceeds 1%, hence considering a larger area at risk than results from the deterministic approach. This could have very important implications in the context of insurance, liability and litigation [63]. By taking this approach, adaptation is not limited by the uncertainty around future scenarios of risk [64]. However, the question of how much uncertainty people are willing to accept remains unsolved and warrants further research on perceptions of risk and decision making.

In order to improve SLR vulnerability assessments and facilitate their effective implementation for adaptation policies, we suggest further research into extending the presented probabilistic framework approach by incorporating uncertainties in other components of SLR vulnerability assessments, such as sea-level forecasts [28], determination of water levels or estimates of population at-risk. Additionally, in areas where coastal systems are more dynamic, such as high-energy sandy beaches or rapidly accreting mangroves or salt marshes, vulnerability assessments can be improved by replacing the static bathtub mapping by process-based modelling that better incorporates the physics of hydrodynamics and coastal erosion/accretion [65].

## Conclusions

- Uncertainty is inherent to spatial data and spatial analysis and therefore it is of paramount importance to effectively communicate it, particularly when dealing with decision-making in a changing climate.
- The spatially variable and spatially correlated elevation error in LiDAR-derived DEMs can be partially explained by land cover and terrain variables.
- Geostatistical modelling of DEM error takes the spatial correlation in these errors into account, which has a great impact on analyses that include spatial interactions such as bathtub inundation modelling
- Sequential Gaussian simulation allows dealing effectively with the complexities of analysing uncertainty propagation through the spatial analysis process and hence is suitable for the mapping of complex and dynamic phenomena such as inundation due to future SLR, storm surge or a combination of these.
- The presented probabilistic approach can be used in a risk-aversive decision making process by planning for scenarios with different probabilities of occurrence. Being able to quantify the probability of inundation at any location is of great interest from a risk management perspective.
- In this study, results showed that when considering a 1% probability exceedance, the inundated area was approximately 11% larger than the mapped using the deterministic bathtub approach.
- Further research is suggested to address the question of how much uncertainty people are willing to accept and the perceptions of risk on decision making.
- SLR vulnerability assessments can be improved by extending the presented probabilistic framework to including uncertainties in sea-level forecasts, determination of water levels and estimates of population at-risk.
